# Tai Chi Chuan for Subjective Sleep Quality: A Systematic Review and Meta-Analysis of Randomized Controlled Trials

**DOI:** 10.1155/2020/4710527

**Published:** 2020-08-11

**Authors:** Yuhao Si, Cenyi Wang, Heng Yin, Jinghui Zheng, Yang Guo, Guihua Xu, Yong Ma

**Affiliations:** ^1^The First School of Clinical Medicine, Nanjing University of Chinese Medicine, Nanjing 210023, China; ^2^Rangos School of Health Sciences, Duquesne University, Pittsburgh 15282, USA; ^3^School of Physical Education and Sports Science, Soochow University, Suzhou 215021, China; ^4^Department of Traumatology & Orthopedics, Wuxi Affiliated Hospital of Nanjing University of Chinese Medicine, Wuxi 214071, China; ^5^Department of Cardiology, Ruikang Hospital Affiliated to Guangxi University of Chinese Medicine, Nanning 530011, China; ^6^School of Nursing, Nanjing University of Chinese Medicine, Nanjing 210023, China; ^7^Department of Traumatology & Orthopedics, Affiliated Hospital of Nanjing University of Chinese Medicine, Nanjing 210029, China; ^8^College of Basic Medicine, Nanjing University of Chinese Medicine, Nanjing 210023, China

## Abstract

**Background:**

This review aims to investigate the efficacy of Tai Chi Chuan on subjective sleep quality among adults.

**Methods:**

We systematically searched PubMed, Embase, Cochrane Library, Scopus, CNKI (China National Knowledge Infrastructure), and the Wanfang Database from their inception to August 2019 and identified 25 eligible studies that were published in both English and Chinese.

**Results:**

24 out of 25 studies were identified to be high-quality studies according to the PEDro scale. The pooled results confirmed that Tai Chi Chuan elicited moderate improvements in subjective sleep quality (SMD = −0.512, 95% CI [−0.767, −0.257], *P* < 0.001). Notably, Tai Chi Chuan yielded more significant effects on sleep quality among the healthy population (SMD = −0.684, 95% CI [−1.056, −0.311], *P* < 0.001) than the clinical population (SMD = −0.395, 95% CI [−0.742, −0.047], *P*=0.026) and more benefits among the Asian population (SMD = −0.977, 95% CI [−1.446, −0.508], *P* < 0.001) than the American population (SMD = −0.259, 95% CI [−0.624, 0.105], *P*=0.164). After controlling the methodological quality of studies, it has been noted that Asians could achieve the most significant sleep-promoting benefit when Tai Chi Chuan was practiced between 60 and 90 min per session.

**Conclusions:**

Available data implied that subjective sleep quality was improved via Tai Chi training, but more thorough studies must be executed to ascertain our findings and optimize Tai Chi practices accordingly toward various populations.

## 1. Introduction

Sleep plays a critical role in the general health of human beings, accounting for almost 1/3 of our lifetimes [1]. Adequate and comfortable sleep is important for motivation and enthusiasm yielding high energy and a happy mood along with the ability to handle daytime routine tasks. However, poor sleep quality has become a universal issue in modern society, causing insidious physical and psychological disorders in both healthy individuals as well as clinical patients [2, 3]. Recent epidemiological investigations indicated that approximately 25% of adults had sleep complaints, 10%–15% experienced insomnia symptoms along with daytime consequences, and 6%–10% met the diagnostic criteria for insomnia disorder [4–9]. Additionally, some indirect consequences caused by poor sleep quality, such as work absenteeism and reduced productivity, may also lead to increased economic and social burdens. Despite the pervasiveness and burden of the issue, poor sleep quality is usually overlooked and untreated due to evaluation and management barriers. It is estimated that only approximately 15% of adults with diagnosed insomnia or sleep complaints attempt to seek professional consultation [10].

Complementary and alternative approaches have been employed to mitigate sleep disorders for a long time, among which physical exercise draws increasing attention in recent years [11]. As a type of physical exercise rooted in traditional Chinese medicine, Tai Chi Chuan has been practiced to invigorate both physical and mental health and prevent diseases for hundreds of years in China [12]. Nowadays, Tai Chi Chuan is drawing legions of practitioners and followers in western countries [13]. It consists of slow movements, weight shifting, concentration, and meditation. Such movements are thought to improve multiple aspects of body health such as control of breath, calming the mind, increasing musculoskeletal strength, and enhancing balance as well as functions of the autonomic nervous and immune systems [14, 15]. Numerous systematic reviews have been directed as a means of providing high-quality evidence of the role of Tai Chi Chuan in alleviating chronic conditions. Some recent reviews have investigated and supported the use of Tai Chi Chuan exercise for chronic conditions, such as cancer-related symptoms [16], cognition impairment [17], type 2 diabetes mellitus [18], stroke rehabilitation [19], coronary heart disease [20], heart failure [21], osteoporosis [22], hypertension [23], chronic musculoskeletal pain [24], chronic obstructive pulmonary disease [25], osteoarthritis [26], and rheumatoid arthritis [27]. Notably, Tai Chi Chuan training could also be efficacious in reducing fall incidence and maintaining and enhancing lower limb proprioception and gait ability for the aged [28]. Overall, the aforementioned reviews reported moderate to strong evidence of Tai Chi Chuan's positive effects on related chronic conditions. However, the application of Tai Chi Chuan on sleep quality seems to dictate varying results.

Some previous systematic reviews evaluated the effectiveness of Tai Chi Chuan (may be presented as a type of mind-body therapies or meditative movements) on sleep quality [29–33]. However, certain limitations were discovered in the previous systematic reviews. Firstly, several systematic reviews meta-analyzed numerous trials regarding mind-body exercises (e.g., Qigong, Yoga, and Tai Chi Chuan), but Tai Chi Chuan only accounted for a small portion of those included trials, implying a lack of independent assessments and weak evidence. This is not to mention that people with various physical conditions or diseases can respond to Tai Chi exercise in different ways, and no analysis was performed based on different types of physical conditions. Finally, some trials of Tai Chi Chuan published in China may potentially have been missed because of the language barriers during the retrieval process. We conducted a systematic review in 2014 to solely investigate the impact of Tai Chi exercise on sleep quality among older adults [34]. In our previous study, five randomized controlled trials (RCTs) were pooled in the meta-analysis, which yielded weak evidence showing a beneficial impact of Tai Chi exercise on self-rated sleep quality in healthy older adults. Additionally, due to a lack of evidence, the guidelines presented by the American College of Physicians and European Sleep Research Society both suggested that it is necessary for complementary and alternative therapy or exercise to undergo further assessment as a means to judge its usefulness in the treatment of insomnia [35, 36]. Therefore, for further study, the current review aims to evaluate the effects of Tai Chi Chuan for sleep quality while employing more updated rigorous RCTs that covered both healthy and clinical populations. Sleep quality is assessed either subjectively through self-report or by objective measures. Nonetheless, perceived sleep quality does not necessarily correspond with objective sleep measures. The Pittsburgh Sleep Quality Index (PSQI) is one of the most frequently used instruments for measuring subjective sleep quality and has excellent psychometric properties. Therefore, we applied the outcome of subjective sleep quality in this review due to its evaluation convenience and clinical significance [37].

## 2. Methods

### 2.1. Search Strategy

This review was executed according to the guidance in the “Preferred Reporting Items for Systematic Reviews and Meta-Analyses” (PRISMA) statement [38]. The protocol was prospectively registered in the International Prospective Register of Systematic Reviews (PROSPERO) database in March 2019 (registration number: CRD42019129782) and was published in November 2019 [39].

We designed a broad literature search strategy aiming to identify all the eligible RCTs published in the English and Chinese language in peer-reviewed journals. Six electronic databases, including PubMed, Embase, Cochrane Library, Scopus, CNKI, and the Wanfang Database, were searched to identify trials relating to the effectiveness of Tai Chi Chuan for sleep quality from their inception to August 2019. The following search terms were employed in the English databases (PubMed, Embase, Cochrane Library, and Scopus): Tai Chi, Taiji, Tai Chi Chuan, shadowboxing, taijiquan, sleep, sleep quality, sleep complaints, sleep problems, sleep disorders, sleep disturbance, and insomnia. The equivalent Chinese search terms were utilized in Chinese databases. A manual search was also implemented at the library of Duquesne University and the Nanjing University of Chinese Medicine in the event that there were any missing eligible articles.

### 2.2. Study Selection

Studies involved in this review met the following criteria: (a) trial assessed the effects of Tai Chi Chuan for sleep quality; (b) the study design was RCT; (c) participants were adults; (d) the participants practiced Tai Chi Chuan in the experimental group and did not practice Tai Chi Chuan in the control group; (e) the outcome was measured through the PSQI scale; (f) the trial was published in English or Chinese. Studies that had any of the following criteria were excluded: (a) trials that used comprehensive interventions, such as Tai Chi Chuan with Chinese oral medicine or acupuncture; (b) data were unavailable to extract and calculate whether or not the corresponding author was contacted successfully.

Four reviewers were divided into two pairs, and each pair independently (Guo and Zheng; Si and Wang) screened all titles and abstracts in the initial search. After removing duplicated and unrelated articles, the lists of possibly relevant articles from the two pairs were checked against each other by another reviewer (Xu). The fifth reviewer (Xu) compared the results of two pairs and determined a preliminary list. The full-text reading and evaluation of articles from the preliminary list were implemented by two reviewers (Wang and Guo) following the predefined inclusion/exclusion criteria. Any disagreement was discussed by the two reviewers to reach a consensus. If the disagreement remained unresolved, a final decision would be made by a third reviewer (Ma).

### 2.3. Data Extraction and Quality Assessment

The process of data extraction was completed by two reviewers (Si and Wang) and double-checked by a third reviewer (Ma). Subject characteristics and study designs were recorded in a Microsoft Excel document, including information such as first author, publication year, country, average age, geographic population, sample size, physical condition, duration and follow-up, frequency, intensity, Tai Chi Chuan style, control intervention, sleep quality measurement, baseline and outcome data, and adverse events. If such information was not reported in the article, we emailed the corresponding author to request for relevant materials.

Two researchers (Si and Ma) evaluated the methodological quality of the included studies through using the Physiotherapy Evidence Database (PEDro) scale. The PEDro scale consists of 11 items, and each item is rated from 0 to 10 points. A study with 6 points or more was identified as a high-quality study. Several previous studies recommended the PEDro scale to assess the quality of RCTs (mainly in the area of physical therapy) for systematic reviews because of its high validity and reliability [40–43].

### 2.4. Statistical Analysis

#### 2.4.1. Effect Size Calculation

Sleep quality outcomes, in terms of mean and standard deviations (SD), either reported in the study or those that could be obtained from the corresponding author, were included in the meta-analyses. We utilized the changes from baseline scores for continuous data due to inconsistent baselines of eligible trials, being presented as the mean and SD. Moreover, the global PSQI (SD ranged from 0.44 to 5.78) from each study was the score of self-assessment with inevitable confounding factors, and the standardized mean difference (SMD) was used to provide robust evaluation over the unstandardized mean difference in various statistical situations [44]. According to Cohen [45], SMD is also known as the effect size (ES) and can be interpreted by large (ES ≥ 0.8), moderate (0.5 ≤ ES < 0.8), small (0.2 ≤ ES < 0.5), and trivial (ES < 0.2). The heterogeneity was evaluated using the chi-square and *I*^2^ test, which describes the percentage of variability in the effect estimates. *I*^2^ of 0%, 25%, 50%, and 75% implied nil, mild, moderate, and severe heterogeneity, respectively [46]. We chose a random-effects model to be applied for the meta-analysis because the expected diversity of involved subjects and interventions might lead to the possible existence of heterogeneity.

#### 2.4.2. Subgroup Analysis

We conducted two preplanned subgroup analyses to explore the potential explanation for heterogeneity and to test the effects of Tai Chi exercise on sleep quality according to the varying situations. One subgroup analysis was administered based on the different physical conditions of the included adults (good health, stroke, fibromyalgia, cancer, arthritis, depression, chronic kidney disease, and heart disease). The other one was performed in accordance with geographic populations (Asian, American, and European) in the trials that enrolled healthy individuals.

#### 2.4.3. Meta-Regression and Additive Models

The source of heterogeneity was explored utilizing a random-effects meta-regression. Some characteristics of the recruited participants, as well as the study design and execution, were also explored in addition to the Tai Chi exercise movements. Specifically, aspects such as average age, sample size, control intervention (treated and nontreated intervention), physical condition (healthy and illness), disease (good health, stroke, fibromyalgia, cancer, depression, arthritis, chronic kidney disease, and heart disease), supervision (supervised, unsupervised, and both), methodological quality of trials (PEDro score), Tai Chi Chuan style (Yang style, Chen style, and Tai Chi Ball), treatment duration (week), training intensity (min/session), frequency (times/week), and geographic population (American, Asian, and European) were entered as variables. All these covariables were input and analyzed one by one. The final covariables were selected depending on their impact on sleep quality response to Tai Chi exercise, which were tested by the statistical significance of *P* < 0.05 combined with adjusted *R*^2^. For the change of PSQI scores, additive models were yielded from all the possible combinations of the final covariables to determine the most significant potential sleep-promoting effects from Tai Chi Chuan. Significant continuous variables such as training intensity (each class: 30–60 min; 60–90 min; 90–120 min) and methodological quality of trials (lowest tier, ≤5; middle tier, 6 to 7; highest tier, ≥8) were coded as category variables in additive models applying prior reviews as a guide [47, 48].

#### 2.4.4. Sensitivity Analysis

Aiming to investigate the stability of our meta-analyses' results, we applied two methods in the sensitivity analysis. First, we chose the trim and fill technique to perform a sensitivity analysis in all the reviewed RCTs and in trials that only recruited healthy individuals. Second, the negative control group was chosen over the positive control group when the study consisted of two or more control groups. Consequently, we conducted a sensitivity analysis to examine whether involving different control groups would yield different results. The publication bias was appraised for using the funnel plot as well as statistical tests (Egger's test and Begg's test).

The meta-analyses were performed based on at least two trials utilizing software Stata version 12.0 (available from the website for purchasing: https://www.stata.com/products/). A two-sided *P* < 0.05 was defined as statistically significant.

## 3. Results

### 3.1. Study Selection

**Figure 1:**
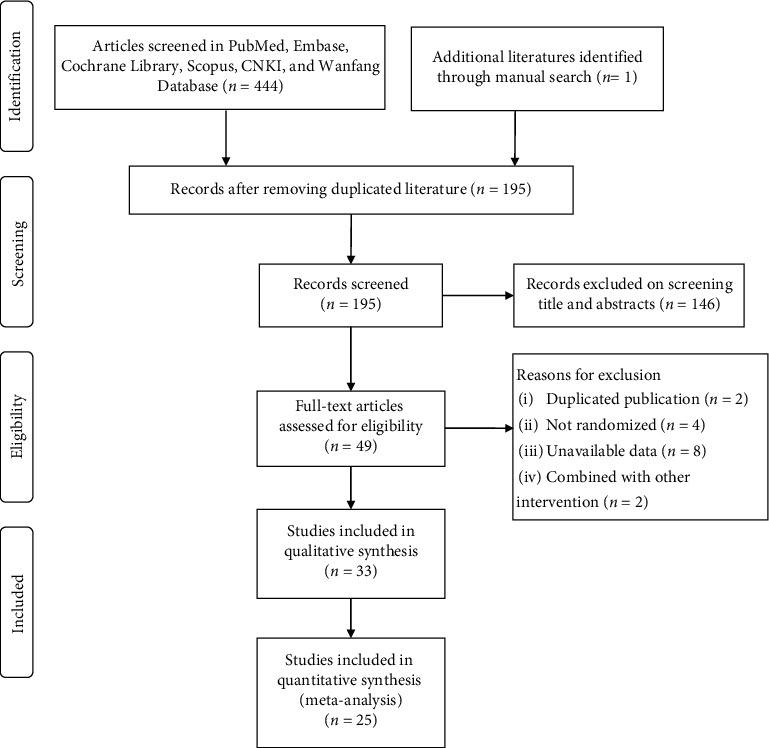
PRISMA flow diagram for screening and identifying eligible studies.

Figure 1 demonstrates the study selection process. A total of 444 studies were considered for inclusion from four English databases and two Chinese databases. We screened 195 relevant studies based on their abstracts and titles after removing duplicates and then excluded 146 studies because they failed to meet the inclusion criteria. We then reviewed 49 full-text articles and identified a total of 25 RCTs that were eligible for this review [49–73], including 16 English articles and nine Chinese articles. The main reasons for exclusion included studies with duplicated publications, those with a nonrandomized design, and studies that did not contain valuable data. Other main reasons included outcomes with non-self-reported measurements or other self-rated scales except PSQI and those combined Tai Chi exercise with other interventions.

### 3.2. Characteristics of Included Studies

The principal characteristics of all included RCTs are shown in Table 1. A total of 1858 adults (average age from 50 to 75 years old) were involved in eligible RCTs with 951 individuals assigned to the Tai Chi Chuan intervention group. The included RCTs were conducted between 2004 and 2018 from six countries (11 trials in the U.S, 10 in China, and one each in Japan, Vietnam, Italy, and Iran, respectively). The treatment duration of the included studies ranged from 8 to 36 weeks, and the follow-up time ranged between 8 and 64 weeks. The frequency of intervention varied widely ranging from once per week to 7 times per week, and every class duration was 30 to 120 min. An estimate was then made that the treatment duration was approximately 40.93 ± 23.22 (*h*, mean ± SD), and each class lasted 57.08 ± 23.60 (min, mean ± SD). Among all the eligible studies, 10 trials evaluated the effects of Tai Chi Chuan for sleep quality in healthy individuals [49–53, 56, 58, 59, 62–68, 71, 73–76]; four in fibromyalgia patients [54, 57, 64, 69, 72, 77, 78]; three in cancer survivors [61, 68, 70, 79–81]; two in patients who experienced stroke [55, 60, 70, 82], heart disease [66, 67, 83, 84], and depression [71, 73, 85, 86]; and one each in people with chronic kidney disease [65] and arthritis [69]. The Tai Chi Chuan intervention in the majority of included studies was subjected to the Yang style [49, 50, 52, 54–61, 63–73]. Chen style [53] and Tai Chi Ball [51, 62] were also adopted in the remaining studies. Notably, the control groups were given diverse interventions such as education, routine daily activity, usual care, waiting list, acupuncture, and some other active exercises. No adverse event was reported in all the included trials.

## 4. Methodological Quality

As illustrated in Table 2, the PEDro scores of the majority (96.00%) of the reviewed trials are 6 points or greater, which reflects a moderate to high literature quality. The most common flaws were that participants and therapists in most of the RCTs were unblinded to the intervention, and 14 studies failed to blind assessors [50, 51, 53, 55, 56, 58, 62–67, 70, 71]. Furthermore, the process of concealed allocation was reported in eight studies [49, 52, 54, 59–61, 68, 72]. Meanwhile, the intention-to-treat analysis was rated positive in seven studies [49, 52, 54, 59, 60, 68, 72] and more than 15% of participants in the five studies dropped out during the intervention [49, 58, 68, 72, 73]. The remaining items were recorded positive in all the involved studies.

### 4.1. Meta-Analysis of the Effects of Tai Chi Chuan on Sleep Quality

**Figure 2:**
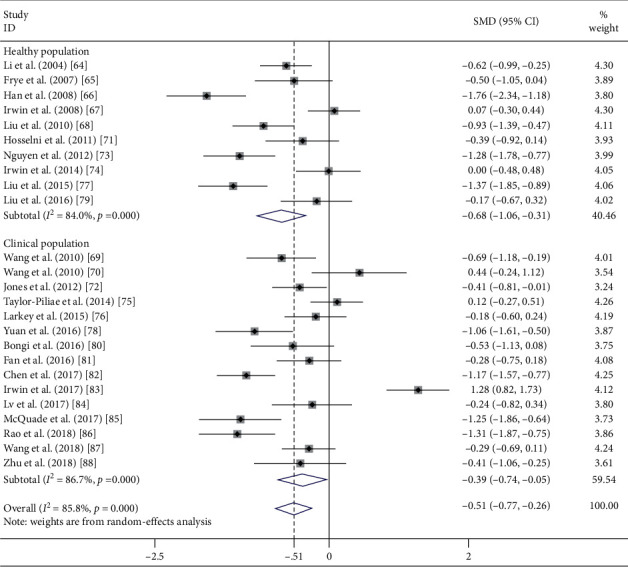
Effect of population condition (healthy and clinical) on subjective sleep quality.

A total of 25 reviewed studies [49–73] were eligible for the meta-analysis which yielded a moderate effect of Tai Chi Chuan in improving subjective sleep quality in comparison to control interventions (SMD = −0.512, 95% CI [−0.767, −0.257], *P* < 0.001; Figure 2). However, a severe statistical heterogeneity was detected (*I*^2^ = 85.8%; *χ*^2^ = 168.45, df = 24, *P* < 0.001).

### 4.2. Impact of Tai Chi Chuan on Sleep Quality in Healthy Adults

Because a substantial amount of heterogeneity was observed, we ran a subgroup analysis on the basis of various conditions of the involved samples (see Figure 2). 10 trials recruited healthy subjects [49–53, 56, 58, 59, 62, 63]. The merged results assumed that Tai Chi Chuan exercise elicited a moderate to large effect in people with good health (SMD = −0.684, 95% CI [−1.056, −0.311], *P* < 0.001) in comparison with the control group. However, significant heterogeneity among studies still existed (*I*^2^ = 84.0%, *χ*^2^ = 56.18, df = 9, *P* < 0.001; Figure 2).

#### 4.2.1. Geographic Population

**Figure 3:**
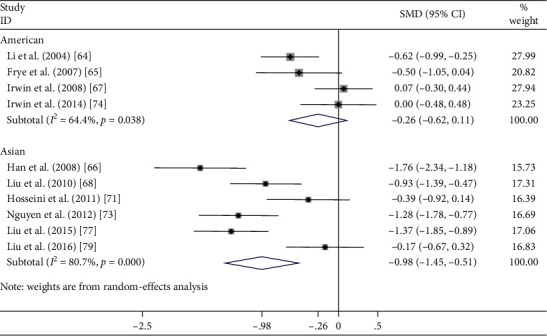
Effect of geographic population (Americans and Asians) on sleep quality in healthy adults.

Preplanned subgroup analyses were conducted according to two geographic populations including 422 Asians [51, 53, 56, 58, 62, 63] and 357 Americans [49, 50, 52, 59] (see Figure 3). The subgroup analysis results indicated that the pooled effects on sleep quality for the six studies recruiting the Asian population were large (SMD = −0.977, 95% CI [−1.446, −0.508], *P* < 0.001) and had severe statistical heterogeneity (*I*^2^ = 80.7%, *χ*^2^ = 25.96, df = 5, *P* < 0.001). In contrast, the subgroup of trials that enrolled the American population did not reveal any significant change of sleep quality (SMD = −0.259, 95% CI [−0.624, 0.105], *P*=0.164), and the results were substantially heterogeneous (*I*^2^ = 64.4%, *χ*^2^ = 8.43, df = 3, *P*=0.038).

### 4.3. Impact of Tai Chi Chuan on Sleep Quality in the Clinical Population

**Figure 4:**
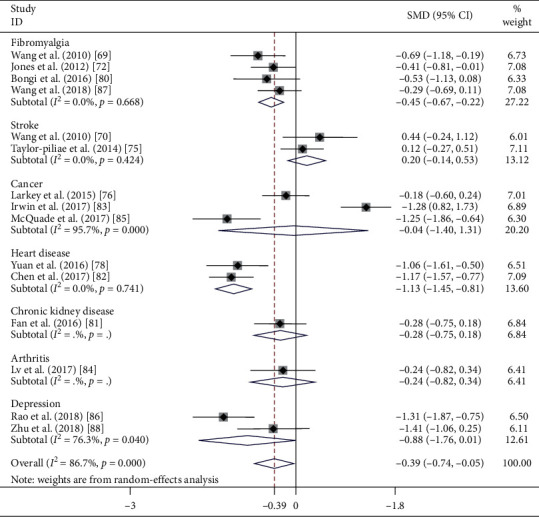
Effect of disease type on sleep quality.

As illustrated in Figure 4, 15 RCTs targeted the clinical population [54, 55, 57, 60, 61, 64–73]. The pooled results reflected that Tai Chi exercise yielded small effects on sleep quality (SMD = −0.395, 95% CI [−0.742, −0.047], *P*=0.026) and the pooled effect size was notably heterogeneous (*I*^2^ = 86.7%, *χ*^2^ = 105.41, df = 14, *P* < 0.001). The subgroup analyses of these 15 trials were tailored on the basis of various disease types.

#### 4.3.1. Stroke

Two studies estimated the effects of Tai Chi Chuan on sleep quality in people who have experienced strokes [55, 60]. In comparison to the control intervention, the aggregated results revealed that Tai Chi Chuan did not significantly improve sleep (SMD = 0.196; 95% CI [−0.143, 0.535]; *P*=0.258). No statistical heterogeneity was present in ESs between studies (*I*^2^ = 0%, *χ*^2^ = 0.64, df = 1, *P*=0.424).

#### 4.3.2. Fibromyalgia

Four RCTs [54, 57, 64, 72] recruited fibromyalgia patients, and the pooled results demonstrated that Tai Chi exercise led to a notable advancement in sleep quality, with a small to moderate effect (SMD = −0.446; 95% CI [−0.674, −0.219]; *P* < 0.001). We found no statistical heterogeneity in ESs among the four studies (*I*^2^ = 0%, *χ*^2^ = 1.56, df = 3, *P*=0.668).

#### 4.3.3. Cancer

Three RCTs tested the impact of Tai Chi Chuan on the sleep quality of cancer survivors [61, 68, 70]. One trial reported that Tai Chi Chuan had a better sleep-promoting impact compared with control interventions [70], but the pooled results did not sustain the positive effects of Tai Chi Chuan in enhancing sleep quality (SMD = −0.041; 95% CI [−1.397, 1.315]; *P*=0.953). The statistical heterogeneity in ESs among studies was extremely severe (*I*^2^ = 95.7%, *χ*^2^ = 46.39, df = 2, *P* < 0.001).

#### 4.3.4. Depression

Two of the reviewed studies recruited subjects with depression [71, 73] and indicated no significant effect on subjective sleep quality (SMD, −0.875; 95% CI [−1.760, 0.010]; *P*=0.053) with severe heterogeneity (*I*^2^ = 76.3%, *χ*^2^ = 4.22, df = 1, *P*=0.040).

#### 4.3.5. Heart Disease

Two studies enrolled patients with heart disease [66, 67, 83, 84]. Among all the subgroup analyses based on diseases, these two trials had the largest pooled effects on sleep disorders (SMD = −1.132; 95% CI [−1.455, −0.809]; *P* < 0.001) and contained no statistically detected heterogeneity (*I*^2^ = 0%, *χ*^2^ = 0.11, df = 1, *P* < 0.001).

### 4.4. Meta-Regression and Additive Models

As shown in Table 3, the results revealed that the methodological quality of included studies significantly correlated with the effects of Tai Chi Chuan for sleep quality, and it was the most important source of heterogeneity with a *P* value of 0.012. In addition, geographic population (*P*=0.038) and training intensity (*P*=0.026) were also notable factors affecting sleep quality response to Tai Chi Chuan training. Generally, these three covariables (methodological quality, geographic population, and training intensity) could explain the 28.96%, 15.09%, and 17.22% of the heterogeneity source in sleep quality response to Tai Chi Chuan training, respectively.

Notably, it has been observed that Tai Chi Chuan elicited greater sleep quality improvements in the Asian population (*P* < 0.001) than in the American population (*P*=0.226). It has also yielded more improvements among subjects who engaged in Tai Chi exercise of low (*P* < 0.002) or moderate (*P* < 0.001 training intensity than vigorous intensity (*P* < 0.579). Furthermore, sleep quality enhancements were more significant among studies that belonged in the middle tier (*P* < 0.001) of methodological quality assessed by the PEDro scale than those in the highest tier (*P*=0.839). The additive statistical models illustrated that after controlling the factors of geographic population and methodological quality of studies, participants could achieve the most considerable sleep-promoting benefits when Tai Chi Chuan was practiced between 60 and 90 min per session (SMD, −0.795; 95% CI [−1.246, −0.344]; *P*=0.001).

### 4.5. Publication Bias

**Figure 5:**
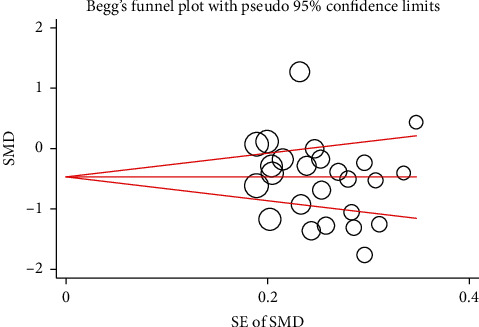
Funnel plot for publication bias. SMD: standardized mean difference; SE: standard error.

A funnel plot was completed based on 25 RCTs [49–73]. According to the visual assessment, no significant evidence of publication bias was identified. This is due to the fact that the spots were roughly symmetric, although several studies seemed to be remarkable outliers (see Figure 5). The results of Begg's test (*P*=0.183) and Egger's test (*P*=0.660) also noted the consistency and registered no significant publication bias in the meta-analysis results.

### 4.6. Sensitivity Analysis

Severe heterogeneity was detected among studies, which indicated that it was necessary to conduct the sensitivity analysis. Firstly, four out of the 25 reviewed studies [50, 59, 60, 70] had two control groups, and the negative control group was chosen over the positive control group in the preplanned meta-analyses. As a result of this, we directed a sensitivity analysis to determine if selecting substituted control groups would yield varying outcomes. A similar result of the sensitivity analysis (SMD = −0.497, 95% CI [−0.773, −0.222], *P* < 0.001) was found in comparison to the preplanned meta-analysis (SMD = −0.512, 95% CI [−0.767, −0.257], *P* < 0.001). Secondly, the trim and fill analysis was applied in a random-effects model among all the 25 included trials [49–73], and this was also applied in the 10 trials [49–53, 56, 58, 59, 62, 63] that solely recruited healthy subjects. The results of the trim and fill technique demonstrated that no trial had been trimmed or filled in both study selection methods, which suggests good stability of the meta-analysis results.

## 5. Discussion

The current review investigated the evidence of the effects of Tai Chi exercise on subjective sleep quality in adults. We conducted the meta-analysis utilizing the extracted data from 25 RCTs with moderate to high quality. The central findings of all the meta-analyses are that (1) Tai Chi Chuan training can yield a moderate effect in improving sleep quality in both healthy and clinical populations; (2) Tai Chi exercise is able to elicit greater sleep-promoting benefits in the healthy population than in the clinical population and more benefits among Asians than in Americans; (3) after controlling methodological quality and geographic population, the optimal Tai Chi training intensity for the Asian population is identified to be between 60 and 90 min per session.

We generally detected that there was a moderate effect (SMD = −0.512, 95% CI [−0.767, −0.257], *P* < 0.001, *k* = 25) of Tai Chi Chuan on subjective sleep quality in all the included samples. Three previous systematic reviews and meta-analyses also evaluated the impact of Tai Chi Chuan training on sleep quality (healthy and clinical populations were pooled together). Our results were more conservative than those reported by Wu et al. [29] (SMD = −0.64, 95% CI [−0.97, −0.30], *P* < 0.01, *k* = 9) and Raman et al. [33] (SMD = −0.89, 95% CI [−1.50, −0.28], *P* < 0.001, *k* = 8) and more progressive than those in the study of Wang et al. [32] (SMD = −0.35, 95% CI [−0.63, −0.07], *P*=0.016, *k* = 12). There appeared to be a lack of consistency between current and previous results, but it should be noted that our findings may be more comprehensive to their results for two main reasons: (a) the number of eligible trials in these three systematic reviews and meta-analyses seemed insufficient, and no study published in Chinese was involved in the reviews of Wang et al. [32] and Raman et al. [33]. On the contrary, we compiled and meta-analyzed more updated RCTs published in both English (*k* = 16) and Chinese (*k* = 9) which implies a more considerable amount of evidence. (b) Wu et al. [29] included two trials that were published in Chinese in their review. However, the two trials combined Tai Chi Chuan with other interventions (e.g., music therapy), which might have resulted in an overestimation of the aggravated ESs [74, 82]. Therefore, our results may have yielded a more precise representation of the effects of Tai Chi Chuan on sleep quality in both healthy and clinical populations.

Furthermore, we also found that there was a moderate to large effect in nonclinical population (SMD = −0.684, 95% CI [−1.056, −0.311], *P* < 0.001, *k* = 10) and a small effect among the clinical population (SMD = -0.395, 95% CI [−0.742, −0.047], *P*=0.026, *k* = 15). To our knowledge, however, no systematic review and meta-analysis which separately evaluates the sleep-promoting effects of Tai Chi exercise in healthy and clinical populations has been conducted. Consequently, we can only compare the current results with the previous study provided by our research group at the Nanjing University of Chinese Medicine [34]. The previous review meta-analyzed five eligible RCTs that recruited healthy older adults, and the results indicated that Tai Chi Chuan elicited significant sleep improvements in older people (SMD = −0.87, 95% CI [−1.25, −0.49], *P* < 0.01, *k* = 5), which were more progressive than our current findings. The differences between the previous and current results are most likely because only five RCTs were included in the previous study (all included in this review) which may have resulted in a lack of accuracy and representation. In addition, Yang et al. [79] conducted a systematic review and meta-analysis of five RCTs to evaluate the impact of exercise (moderate-intensity aerobic exercise or high-intensity resistance exercise) for self-reported sleep quality in healthy adults (288 participants) and found a nearly moderate effect size of 0.47 (95% CI [0.08, 0.86], *k* = 5), which was smaller than what was reported by us for Tai Chi Chuan. Unfortunately, we were unable to make any comparison with the previous results in relation to the aggregated effects of Tai Chi exercise on sleep quality among the clinical population as we did not identify any qualified review.

The eligible RCTs in our review enrolled subjects with various physical conditions, including good health, stroke, fibromyalgia, heart disease, cancer, arthritis, depression, and chronic kidney disease. Therefore, one subgroup analysis was administrated based on various physical conditions, the results of which indicated that Tai Chi exercise was more effective in improving sleep quality among healthy adults (*k* = 10) as well as patients with fibromyalgia (*k* = 4) and heart disease (*k* = 2). Nonetheless, only moderate evidence of the impact of Tai Chi exercise on sleep promotion among healthy adults could be preliminarily proved since the results of fibromyalgia and heart disease were yielded by fewer involved trials. Moreover, the other subgroup analysis was to compare the various sleep responses to Tai Chi training between healthy Asian and American populations. The pooled results revealed a demonstrable difference between the two geographic populations that Asian subjects gained statistically significant sleep quality benefits from Tai Chi Chuan, while the Americans did not. One potential cause that may explain the results is as follows: as is known to all, Tai Chi Chuan originates from ancient China and has been developed by the various generations of Chinese people [33]. Simply put, Tai Chi Chuan is fundamentally a tailor-made form of exercise and lifestyle therapy for the body constitutions of Chinese people, which resembles populations from other Asian countries (e.g., Korea and Vietnam) rather than the American population.

Tai Chi exercise was proved efficacious in mitigating poor sleep quality in numerous previous studies [29–32, 34], but its training intensity, frequency, duration, and styles varied immensely. Consequently, a particular objective of this review was to identify the combination of Tai Chi Chuan elements that yields the most effective enhancement of sleep quality by utilizing additive models. After controlling the methodological quality of studies, the most practical and clinically relevant finding in this review was that Tai Chi exercise could yield the most significant sleep quality improvement when it was practiced between 60 and 90 min per session among Asian participants. Several previous systematic reviews recommended the prescriptions of other Tai Chi Chuan elements (style, treatment duration, and frequency) in chronic conditions [18, 26, 75], but no review found any better option of the training intensity (session time) so far. Consequently, our findings might be novel and encouraging with respect to the use of Tai Chi Chuan for chronic conditions.

### 5.1. How Tai Chi Chuan Elicits Sleep Quality Improvements

Although a number of studies have supported the application of Tai Chi Chuan on adults with poor sleep quality [29–34], the exact biological mechanism remains unclear. It appears that Tai Chi Chuan can enhance the mind-body connection and control through integrating physical, emotional, psychosocial, spiritual, and behavioral elements. The multiple components of Tai Chi Chuan may act through intermediate pathways (e.g., neuroendocrine, immune function, and neurochemical) to improve psychological and behavioral factors associated with poor sleep quality, such as anxiety, depression, and chronic pain [52, 76, 83]. In addition, Montgomery and Dennis [77] claimed that Tai Chi practitioners had higher energy expenditure during the day; thus, they required greater rest at night. Nevertheless, Irwin et al. [52] argued that there were no significant changes in the physical energy expenditure yielded by Tai Chi Chuan, and the sleep enhancement seemed to be independent of energy expenditure. Chao et al. [87] found that the average exercise intensity of Tai Chi Chuan was 3.1 MET (metabolic equivalent). Therefore, Tai Chi Chuan is a low-intensity exercise. As shown in the studies reviewed here, we believe that the gentle movements, slow rhythm, deep diaphragmatic breathing, and emotional relaxation of Tai Chi Chuan can improve the feelings of well-being and ameliorate mental state, which may be the critical factors to the sleep quality improvement. However, the specific mechanism by which Tai Chi Chuan improves sleep quality and other outcomes in the context of these various health conditions are needed to be further studied.

### 5.2. Tai Chi Chuan and Cognitive Behavioral Therapy

Our findings support the perspective of applying Tai Chi Chuan as a complementary and alternative therapy for poor sleep quality to some degree because the ESs of the changing global PSQI scores elicited by Tai Chi Chuan are approximately equivalent to the ESs of those reported for cognitive behavioral therapy (CBT). Notably, CBT is currently highly recommended as the first-line treatment by both the American College of Physicians as well as the European Sleep Research Society in the management of insomnia [35, 36, 80, 84]. Because CBT has been proven effective for insomnia and is currently widely used, the following question is: why we still need Tai Chi Chuan to treat people with poor sleep quality? First, CBT is an intensive form of intervention that requires proper administration and supervision by highly trained clinicians [88]. Thus, the CBT medical resources may be low cost-efficient and nonpragmatic in usual medical institutions. This is particularly true for clients with a moderate level of sleep complaints rather than syndromal insomnia [52]. However, Tai Chi Chuan can be learned and practiced in a relaxed manner both indoors and outdoors and can even be directed by a video tutorial at home. Second, people with poor sleep quality may stay motivated with Tai Chi Chuan training as it can be practiced in a group-based form and offers social support and enjoyment [81]. Finally, Tai Chi Chuan provides not only mental health improvements (e.g., dementia, depression, and insomnia) but also positive effects on various common diseases (e.g., osteoarthritis, Parkinson's disease, and chronic obstructive pulmonary disease) [85]. Despite the considerable positive benefits discussed, whether Tai Chi Chuan to some extent is superior to universally recommended CBT is still open for dispute until further studies validate the current findings.

### 5.3. Analysis of High Heterogeneity among Included Trials

A moderate to high level of heterogeneity was observed either in our review or in the previous systematic reviews and meta-analyses [29, 32–34]. Although we applied more restrictive search strategies that only recruited RCTs with PSQI as the outcome measurement, the heterogeneity of meta-analysis results did not decline as more Chinese and English articles and several conditions of participants were involved. Two preplanned subgroup analyses seemed to explain the high heterogeneity to some degree. On the one hand, the heterogeneity decreased to a moderate level (*I*^2^ = 64.6%, *P*=0.038) among studies recruiting healthy Americans in the subgroup of the geographic population. On the other hand, the disease-based subgroup eliminated the heterogeneity among studies that recruited participants with stroke, fibromyalgia, and heart disease, respectively (*I*^2^ = 0%). In order to examine potential contributors to the severe heterogeneity among trials, we then performed the meta-regression of the ESs for global PSQI scores, and several moderators of the sleep quality responses to Tai Chi Chuan were identified: (a) sleep quality promotions were most considerable among trials that prescribed Tai Chi exercise for 60 to 90 min per session, followed by trials that practiced Tai Chi for 30 to 60 min per session; (b) sleep enhancements were more effective among trials that recruited the Asian population than trials with the American population; (c) sleep quality improvements were more significant in trials that were ranked in the middle tier of methodological quality than trials that ranked in the highest tier of methodological quality. Interestingly, among all the tested moderators, physical condition (fitness and illness) and disease (good health, stroke, fibromyalgia, cancer, depression, arthritis, chronic kidney disease, and heart disease) did not manifest any statistical significance in the regression model. This could possibly be attributed to the fact that there were so few trials in each disease (stroke: *k* = 2, fibromyalgia: *k* = 4, cancer: *k* = 3, chronic kidney disease: *k* = 1, heart disease: *k* = 2, arthritis: *k* = 1, and depression: *k* = 2) which led to insufficient statistical power for difference detection. Instead, the methodological quality of studies was the largest source of heterogeneity among the studies reviewed, the exact explanation of which was unclear. Nevertheless, we observed that trials with moderate and lower methodological quality tended to have been implemented in Asia (*ρ* = −0.743, *P* < 0.001) and consisted of a smaller sample size (*ρ* = 0.543, *P*=0.005) (see Supplementary Data Table S2). More notably, the results of the meta-regression analyses demonstrated that the geographic population was also a cofounder of the heterogeneity identified among studies. This finding suggests that the sleep quality of Asian, American, and European populations may respond to Tai Chi Chuan in different ways. Therefore, it has been identified that the integrated physical, behavioral, spiritual, and psychosocial elements of Tai Chi exercise warrant modification and localization according to the specific constitutions of different geographic populations. Finally, intensity(session time), frequency, and treatment duration were noted to be the three main components of Tai Chi Chuan intervention that were the most influential moderators in our review. However, our analysis of the current data extracted from RCTs revealed that the Tai Chi Chuan training intensity was the only component found that affected sleep quality response to Tai Chi Chuan exercise. Therefore, when physicians and therapists prescribe Tai Chi Chuan exercise for their clients with poor sleep quality or insomnia, training intensity should be emphasized as the first element to be determined according to the health status of clients. Subsequently, the frequency and treatment duration of exercise can be adjusted accordingly based on intensity.

### 5.4. Strengths: Comparison to Previous Systematic Reviews and Meta-Analyses

Some results obtained by this review were consistent with several previous systematic reviews and meta-analyses in that Tai Chi Chuan may be considered beneficial for insomnia [29–34]. However, only two of the previous systematic reviews and meta-analyses regarded Tai Chi Chuan as their main study subject [33, 34], and one of the two studies was directed and supervised by one member (Xu) of our current workgroup [34]. The four remaining systematic reviews and meta-analyses aimed to investigate the evidence of meditative movement interventions or mind-body therapies on sleep quality [29–32]. Two of the four systematic reviews utilized the meta-analysis [29, 32] while the other two used the narrative synthesis [30, 31]. There were nine out of 14 included trials that were related to Tai Chi Chuan intervention in the review of Wu et al. [29], seven out of 112 in the review of Neuendorf et al. [30], nine out of 17 in the review of Wang et al. [31], and 12 out of 49 in the review of Wang et al. [32], respectively.

This current research remarkably differs from the previous one. The latest systematic review and meta-analysis conducted by Wang et al. [32] was discussed as an example to obtain more detailed information concerning the differences between previous systematic reviews and this review. Wang et al. [32] estimated the impact of mind-body therapies on insomnia in both aged healthy and clinical populations. However, some disadvantages were observed which are listed as follows: (a) the reviewed studies involved interventions of Tai Chi Chuan, yoga, meditation, Qigong, and Qigong Tai Chi but only 12 articles were related to Tai Chi Chuan (total: 40); (b) Qigong Tai Chi only consists of the practice of Tai Chi Chuan; thus, Qigong Tai Chi and Tai Chi Chuan were naturally the same and did not warrant a separate evaluation; (c) no pooled results of Tai Chi exercise were reported since no tailored subgroup analysis of Tai Chi Chuan was performed independently; (d) no planned subgroup analysis was directed in terms of the different kinds of physical conditions or diseases; this typically contributed to substantial heterogeneity and sometimes inaccurate results; and (e) Tai Chi Chuan is a traditional Chinese martial art but only English studies were included. Given such limitations, the effects of Tai Chi Chuan on sleep quality remained vague and inconclusive.

On the contrary, the current work recruited a total of 25 updated RCTs published in English and Chinese. We focused on assessing the impact of Tai Chi Chuan on sleep quality and conducted subgroup analyses according to the various physical conditions and geographic populations. Additionally, meta-regression was implemented as a means to explore the possible sources of the high level of heterogeneity. Meanwhile, additive models were also applied to identify the optimal combination of Tai Chi Chuan elements on sleep quality. Moreover, we employed sensitivity analysis methods to test the stability of the meta-analysis results and examined the publication bias in both visual and statistical manners. More importantly, this review compiled the clinically relevant elements of Tai Chi Chuan intervention, which could guide future studies. As a result of all such factors, our review provided more precise meta-analysis results and stronger evidence for Tai Chi Chuan on subjective sleep quality in comparison with previous reviews.

### 5.5. Limitations

The primary limitations of the current review are listed as follows: (a) high heterogeneity existed among included RCTs; this is likely attributed to the fact that data were extracted from participants with various populations and physical conditions; (b) there was an insufficient quantity of reviewed trials in some disease categories (heart disease, depression, arthritis, stroke, and cancer); this may have contributed to weak evidence or even meaningless conclusions; (c) the included trials consisted of inconsistent Tai Chi Chuan components (training intensity, frequency, and treatment duration) which may have influenced the pooled results.

Another limitation is that we choose the global PSQI scores as the sole parameter for sleep quality outcomes. It should be noted that PQSI is a widely translated and employed self-reporting questionnaire, which plays a significant role in sleep status evaluation in both research and medical settings [78, 86]. It contains multiple notable advantages such as high cost-effectiveness, easy implementation, and high patient compliance allowing clinicians and researchers to save considerable time [37]. As a result, PSQI is often employed in sleep-related studies. However, Manzar et al. [89] found the multiple PSQI factor structures for sleep quality appraisal in both clinical and nonclinical settings to be doubtful and in possible need of further validation. Besides, sleep quality changes in insomniacs comorbid with psychiatric and medical conditions which could also be embodied through other disease-specific approaches (e.g., applying an electrocardiogram-based spectrogram method to evaluate sleep quality in patients with chronic heart disease) [90]. Nevertheless, we could not assess these disease-specific outcomes as they are not universally utilized in the RCTs with regard to Tai Chi exercise for sleep quality.

Although the meta-regression and additive models identified a clinically meaningful proportion of each factor in ESs, a large amount of heterogeneity of Tai Chi Chuan sleep-promoting effects unsurprisingly remained unexplained. In addition to the factor of geographic population, training intensity, and methodological quality, eight other possible sources of heterogeneity were examined and found to be not statistically significant on the sleep quality responses to Tai Chi exercise (see Supplementary Data Table S1). Otherwise, some researchers from the reviewed Tai Chi Chuan RCTs might have omitted some detailed intervention information (e.g., when was Tai Chi Chuan practiced during the day?) in their studies which were likely to have some objective reasons (e.g., space limitation). This concern could militate against the implementation of our data extraction and analysis. For instance, we were unable to investigate some clinically relevant moderators due to inadequate disclosure, such as the Tai Chi Chuan postures (*k* = 4, 16.0%) and the assessment of participant fatigue after Tai Chi Chuan training (*k* = 3, 12.0%). Consequently, it is necessary for our current results to be interpreted with caution in the context of the aforementioned limitations.

## 6. Conclusion

In conclusion, this present review suggests that Tai Chi Chuan exercise has a moderate beneficial impact on subjective sleep quality among adults. Sleep quality enhancements of this level rival those reported from CBT, the initial nonpharmacological therapy for adults with insomnia. It also reflects that the current authoritative guidelines may include Tai Chi Chuan as a recommended choice of complementary and alternative treatments. Additionally, we discovered that when practiced between 60 and 90 min per session, Tai Chi Chuan can elicit the most considerable sleep-promoting effects among Asians. However, further studies are necessary as a means to optimize other Tai Chi Chuan components in accordance with the particular characteristics and aspects in different populations. The evidence of the effects of Tai Chi exercise on sleep quality among the clinical population appears to be weak. Consequently, more comprehensive and randomized trials of Tai Chi Chuan for subjective sleep quality need to be implemented in order to ascertain the present findings.

## Figures and Tables

**Table 1:** Characteristics of included studies.

Author, year, country	Primary report	Sample size, mean age (years)	Follow-up (weeks)	Outcome measurements	Experimental group intervention (*n*)	Training program	Control group intervention (*n*)	Treatment program
Li et al. (2004), USA	Healthy	118, 75.21	NR	PSQI	8-form Yang style Tai Chi (*n* = 62)	60 min class program, 3 times each week for 24 weeks	Low-impact exercise (*n* = 56)	60 min class program, 3 times each week, for 24 weeks
Frye et al. (2007), USA	Healthy	84, 69.2	14	PSQI	10-form Yang style Tai Chi (*n* = 31)	60 min class program, 3 times each week for 12 weeks	No exercise (*n* = 23)	NR
Han et al. (2008), China	Healthy	64, NR (55 years and older)	NR	PSQI	Tai Chi Ball (*n* = 32)	45 min class program, 3 times each week for 36 weeks	Aerobic exercise (*n* = 32)	45 min class program, 3 times each week for 36 weeks
Irwin et al. (2008), USA	Healthy	112, 60.65	25	PSQI	Yang style Tai Chi (*n* = 59)	40 min class program, 3 times each week for 16 weeks	Health education (*n* = 53)	40 min class program, 3 times each week, for 16 weeks
Liu et al. (2010), China	Healthy	82, 66.03	NR	PSQI	Yang style Tai Chi (*n* = 43)	30 min class program, 5 times each week for 8 weeks	No exercise (*n* = 39)	NR
Wang et al. (2010), USA	Fibromyalgia	66, 50.10	24	PSQI	Classic Yang style Tai Chi (*n* = 33)	60 min class program, 2 times per week +20 min home-based program every day, for 12 weeks	Wellness education (*n* = 33)	60 min class program, 2 times each week, for 12 weeks
Wang et al. (2010), Japan	Stroke	34, NR (50 years and older)	NR	PSQI	Classic Yang style Tai Chi (*n* = 17)	50 min class program, 1 time each week for 12 weeks	Rehabilitation program (*n* = 17)	80 min class program, 1 time each week, for 12 weeks
Hosseini et al. (2011), Iran	Healthy	62, NR (65 years and older)	NR	PSQI	Yang style Tai Chi (*n* = 27)	5 min at the first session and 5 min added in each following week, 3 times each week for 12 weeks	Routine daily activities (*n* = 29)	NR
Jones et al. (2012), USA	Fibromyalgia	98, 54.00	NR	PSQI	8-form Yang style Tai Chi (*n* = 51)	90 min class program, 2 times each week for 12 weeks	Education intervention (*n* = 47)	90 min class program, 2 times each week, for 12 weeks
Nguyen et al. (2012), Vietnam	Healthy	96, 68.90	NR	PSQI	24-form Yang style Tai Chi (*n* = 48)	60 min class program, 2 times each week for 24 weeks	Routine daily activities (*n* = 48)	NR
Irwin et al. (2014), USA	Healthy	98, 65.33	64	PSQI	Yang style Tai Chi (*n* = 48)	120 min class and home-based program, 1 time each week for 16 weeks	Sleep seminar education (*n* = 50)	120 min class program, 1 time each week, for 16 weeks
Taylor-Piliae et al. (2014), USA	Stroke	97, 69.90	NR	PSQI	Tai Chi easy (*n* = 53)	60 min class program, 3 times each week for 12 weeks	Usual care (*n* = 48)	Weekly phone calls, for 12 weeks
Larkey et al. (2015), USA	Breast cancer	87, 58.80	12	PSQI	Yang style Tai Chi (*n* = 42)	30 min home-based program, 5 times each week for 12 weeks	Sham Qigong (*n* = 45)	30 min home-based program, 5 times each week, for 12 weeks
Liu et al. (2015), China	Healthy	84, 63.30	NR	PSQI	Tai Chi Ball (*n* = 44)	60 min class program, 3 times each week for 18 weeks	No exercise (*n* = 40)	NR
Yuan et al. (2016), China	Heart failure combined with depression	60, 66.90	NR	PSQI	24-form Yang style Tai Chi (*n* = 32)	30 min class program, 5 times each week for 12 weeks	Usual care (*n* = 30)	NR
Liu et al. (2016), China	Healthy	63, 66.05	8	PSQI	24-form Yang style Tai Chi (*n* = 32)	60 min class program, 5 times each week for 16 weeks	No exercise (*n* = 31)	NR
Bongi et al. (2016), Italy	Fibromyalgia	44, 52.24	NR	PSQI	Unreported style Tai Chi (*n* = 22)	60 min class program, 2 times each week for 16 weeks	Educational Session (*n* = 22)	No session
Fan et al. (2016), China	Hemodialysis patients	71, 51.23	NR	PSQI	24-form Yang style Tai Chi (*n* = 34)	20 min class program, 2 times each week for the first 12 weeks and 45 min home-based program, 3 times each week for the next 12 weeks	Usual care (*n* = 37)	NR
Chen et al. (2017), China	Coronary heart disease	115, 60.87	NR	PSQI	24-form Yang style Tai Chi (*n* = 57)	60 min class program, 3 times each week for 12 weeks	Usual care (*n* = 58)	NR
Irwin et al. (2017), USA	Breast cancer	90, 59.84	60	PSQI, AISI	Yang style Tai Chi (*n* = 45)	120 min class program, 1 time each week for 12 weeks	Cognitive behavioral therapy (*n* = 45)	120 min class program, 1 time each week for 12 weeks
Lv et al. (2017), China	Arthritis	46, 64.57	NR	PSQI	8-form Yang style Tai Chi (*n* = 23)	60 min class program, 3 times each week for 24 weeks	Wellness education (*n* = 23)	60 min class program, 2 times each week for 24 weeks
McQuade et al. (2017), USA	Prostate cancer	52, 63.60	12	PSQI, 18-item self-rated questionnaire	8-form Yang style Tai Chi (*n* = 26)	40 min class program, 1 time each week for 6 to 8 weeks	Wait list (*n* = 24)	NR
Rao et al. (2018), China	Depression	60, 54.62	NR	PSQI	24-form Yang style Tai Chi (*n* = 30)	60 min class program, 5 times each week for 8 weeks	Acupuncture (*n* = 30)	30 min treatment, 5 times each week for 8 weeks
Wang et al. (2018), USA	Fibromyalgia	111, 52.10	52	PSQI	Classic Yang style Tai Chi (*n* = 36)	60 min class program, 2 times each week for 24 weeks	Aerobic exercise (*n* = 75)	60 min class program, 2 times each week for 24 weeks
Zhu et al. (2018), China	Depression	80, NR	NR	PSQI	24-form Yang style Tai Chi (*n* = 42)	60 min class program, 5 times each week for the first 12 weeks and 60 min class program, 3 times each week for the next 12 weeks	Setting up exercises to radio music (*n* = 38)	4 min and 45 s class program, 5 times each week for the first 12 weeks and 3 times each week for the next 12 weeks

PSQI, Pittsburgh Sleep Quality Index; NR, no report; AISI, Athens Insomnia Severity Index.

**Table 2:** Assessment of methodological quality for included trials based on the PEDro scale.

Author, year	Eligibility criteria	Random allocation	Concealed allocation	Similar at baseline	Subjects blinded	Therapists blinded	Assessors blinded	<15% dropouts	Intention-to-treat analysis	Between-group comparisons	Point measures and variability data	Total
Li et al. (2004)	1	1	1	1	0	0	1	0	1	1	1	8
Frye et al. (2007)	1	1	0	1	0	0	0	1	0	1	1	6
Han et al. (2008)	1	1	0	1	0	0	0	1	0	1	1	6
Irwin et al. (2008)	1	1	1	1	0	0	1	1	1	1	1	9
Liu et al. (2010)	1	1	0	1	0	0	0	1	0	1	1	6
Wang C et al. (2010)	1	1	1	1	0	0	0	1	1	1	1	8
Wang W et al. (2010)	1	1	0	1	0	0	1	1	0	1	1	7
Hosseini et al. (2011)	1	1	0	1	0	0	0	1	0	1	1	6
Jones et al. (2012)	1	1	0	1	0	0	1	1	0	1	1	7
Nguyen et al. (2012)	1	1	0	1	0	0	0	0	0	1	1	5
Irwin et al. (2014)	1	1	1	1	0	1	1	1	1	1	1	10
Taylor-Piliae et al. (2014)	1	1	1	1	0	0	1	1	1	1	1	9
Larkey et al. (2015)	1	1	1	1	1	0	1	1	0	1	1	9
Liu et al. (2015)	1	1	0	1	0	0	0	1	0	1	1	6
Yuan et al. (2016)	1	1	0	1	0	0	0	1	0	1	1	6
Liu et al. (2016)	1	1	0	1	0	0	0	1	0	1	1	6
Bongi et al. (2016)	1	1	0	1	0	0	0	1	0	1	1	6
Fan et al. (2016)	1	1	0	1	0	0	0	1	0	1	1	6
Chen et al. (2017)	1	1	0	1	0	0	0	1	0	1	1	6
Irwin et al. (2017)	1	1	1	1	0	0	1	0	1	1	1	8
Lv et al. (2017)	1	1	0	1	0	0	1	1	0	1	1	7
McQuade et al. (2017)	1	1	0	1	0	0	0	1	0	1	1	6
Rao et al. (2018)	1	1	0	1	0	0	0	1	0	1	1	6
Wang et al. (2018)	1	1	1	1	0	0	1	0	1	1	1	8
Zhu et al. (2018)	1	1	0	1	0	0	1	0	0	1	1	6

0 = does not meet the criteria; 1 = meets the criteria.

**Table 3:** Meta-regression and additive models: sleep quality change response to Tai Chi exercise.

Moderator category/level	*β*	Adjusted *R*^2^ (%)	*P* ^1^ value	Effect size (95% CI)	*P* ^2^ value
Geographic population	−0.563	15.09	0.038		
American (*k* = 11)				−0.213 (−0.558, 0.132)	0.226
Asian (*k* = 13),				−0.778 (−1.097, −0.460)	<0.001
European (*k* = 1)				−0.527 (−1.128, 0.075)	0.086
Methodological quality (score)	−0.675	28.96	0.012		
Lowest tier, ≤5 (*k* = 1)				−1.276 (−1.781, −0.770)	<0.001
Middle tier, 6 to 7 (*k* = 16)				−0.718 (−0.983, −0.453)	<0.001
Highest tier, ≥8 (*k* = 8)				−0.041 (−0.441, 0.385)	0.839
Training intensity (min/session)	−0.930	17.22	0.026		
Low, 30 ≤ time < 60 (*k* = 10)				−0.596 (−0.975, −0.218)	0.002
Moderate, 60 ≤ time < 90 (*k* = 12)				−0.647 (−0.946, −0.348)	<0.001
Vigorous, 90 ≤ time < 120 (*k* = 3)				0.286 (−0.726, 1.298)	0.579
Additive models:					
(1) Among Asian population; (2) among studies with methodological quality of middle tier (PEDro score: 6 to 7 points).					
Low intensity: 30 ≤ time < 60 (min/session)				−0.674 (−1.215, −0.133)	0.015
Moderate intensity: 60 ≤ time < 90 (min/session)				−0.795 (−1.246, −0.344)	0.001

*β*, regression coefficient; *k*, number of trials; adjusted *R*^2^, the proportion of the source of heterogeneity explained by each moderator; *P*^1^ value, the statistical significance of each moderator in the meta-regression model; *P*^2^ value, the statistical significance of subgroup analyses; effect sizes are generated by subgroup analyses based on the three moderators, respectively.

## Data Availability

All data compiled or analyzed during this study are included in this published article.

## Abbreviations

CBT: Cognitive behavioral therapy
CNKI: China National Knowledge Infrastructure
ES: Effect size
PEDro: Physiotherapy Evidence Database scale
PRISMA: Preferred Reporting Items for Systematic Reviews and Meta-Analyses
PROSPERO: International Prospective Register of Systematic Reviews
PSQI: Pittsburgh Sleep Quality Index
RCTs: Randomized controlled trials
SD: Standard deviation
SMD: Standardized mean difference.

## Supplementary Materials

Supplementary Materials Table S1: meta-regression—the eight covariables that did not show any statistical significance. Table S2: Spearman's correlation analysis—the relationship between each factor and methodological quality.
