# Supplementary material for: Tai Chi Chuan for Subjective Sleep Quality: A Systematic Review and Meta-Analysis of Randomized Controlled Trials
Source: Evid Based Complement Alternat Med. 2020 Aug 11;2020:4710527. doi: 10.1155/2020/4710527 (PMC7439202; doi:10.1155/2020/4710527)
Supplement: Supplementary Materials — Table S1: meta-regression—the eight covariables that did not show any statistical significance. Table S2: Spearman's correlation analysis—the relationship between each factor and methodological quality. [file 4710527.f1.docx]

**Table S1**

Meta-regression: The eight covariables that did not show any statistical significance.

| Moderator category/level | Adjusted R^2^ (%) | *P* value |
| --- | --- | --- |
| Sample size | -3.54 | 0.616 |
| Tai Chi Style | 3.65 | 0.387 |
| Supervision | -8.06 | 0.778 |
| Frequency | 5.94 | 0.146 |
| Duration | 0.05 | 0.317 |
| Disease | 5.50 | 0.390 |
| Condition | 0.68 | 0.289 |
| Control intervention | -1.21 | 0.420 |

**Table S2**

Spearman’s correlation analysis: the relationship between each factor and methodological quality

| Factor | ρ | *P* value |
| --- | --- | --- |
| Publication year | -0.111 | 0.598 |
| Sample size | 0.543 | 0.005* |
| Supervision (supervised, unsupervised and both) | 0.288 | 0.162 |
| Trial location (Asia, America, Europe) | -0.743 | < 0.001* |

Note: *, two-sided *P* value < 0.05. ρ, Spearman’s correlation coefficient.
